# Involvement of Prolyl Hydroxylase Domain Protein in the Rosiglitazone-Induced Suppression of Osteoblast Differentiation

**DOI:** 10.1371/journal.pone.0139093

**Published:** 2015-09-29

**Authors:** Ju-Hee Kang, Hyun Jeong Kwak, Hye-Eun Choi, Juyoung Kim, Sangmee Hong, Ok-Hee Kim, Byung Chul Oh, Hyae Gyeong Cheon

**Affiliations:** 1 Department of Pharmacology, School of Medicine, Gachon University, Incheon, Republic of Korea; 2 Department of Molecular Medicine, Gachon University, Incheon, Republic of Korea; 3 Gachon Medical Research Institute, Gil Medical Center, Incheon, Republic of Korea; VCU, UNITED STATES

## Abstract

Rosiglitazone is a well-known anti-diabetic drug that increases insulin sensitivity via peroxisome proliferator-activated receptor γ (PPARγ) activation, but unfortunately it causes bone loss in animals and humans. A previous study showed that prolyl hydroxylase domain protein (PHD) plays a role in rosiglitazone-induced adipocyte differentiation. Based on the inverse relationship between adipocyte and osteoblast differentiation, we investigated whether PHD is involved in the effects of rosiglitazone on osteoblast differentiation. Rosiglitazone inhibited osteoblast differentiation in a concentration-dependent manner, and in parallel induced three PHD isoforms (PHD1, 2, and 3). PHD inhibitors and knockdown of each isoform prevented the inhibitory effects of rosiglitazone on osteoblast differentiation and increased the expression of Runx2, a transcription factor essential for osteoblastogenesis. MG-132, a proteasomal inhibitor also prevented the rosiglitazone-induced degradation of Runx2. Furthermore, both increased PHD isoform expressions and reduced osteoblast differentiation by rosiglitazone were prevented by PPARγ antagonists, indicating these effects were mediated via PPARγ activation. *In vivo* oral administration of rosiglitazone to female ICR mice for 8 weeks reduced bone mineral densities and plasma alkaline phosphatase (ALP) activity, and increased PHD expression in femoral primary bone marrow cells and the ubiquitination of Runx2. Together, this suggests that the rosiglitazone-induced suppression of osteoblast differentiation is at least partly induced via PPARγ-mediated PHD induction and subsequent promotion of the ubiquitination and degradation of Runx2.

## Introduction

Cellular differentiation is a critical requirement for body homeostasis, and is tightly coordinated by the regulation of several transcription factors and intracellular signals. Bone homeostasis is maintained by balance between the activities of osteoblasts and osteoclasts, and imbalance between these cells results in metabolic diseases, such as, osteoporosis and osteopetrosis [1]. Osteoblasts and osteoclasts are derived from different developmental lineages, that is, osteoblast from a mesenchymal lineage [2] and osteoclasts from a hematopoietic lineage [3]. Osteoblasts are responsible for bone formation, which leads to mineralization and further differentiation into osteocytes. Over the last two decades, many factors have been found to regulate osteoblast differentiation. For example, runt-related transcription factor-2 (Runx2), osterix, Msh homeobox-2 (Msx2), bone morphogenetic protein 2 (BMP2), Wnt and Hedgehog have been shown to be required for osteoblastogenesis [4]. Adipocytes also originate from mesenchymal progenitor cells, thus the biological activities of osteoblasts and adipocytes are related. In fact, factors that control osteoblastogenesis have been shown to inhibit adipogenesis, and vice versa [5].

PPARγ belongs to the nuclear receptor family of transcription factors, which regulates fatty acid uptake and adipocyte differentiation [6]. There are two alternative splicing forms of PPARγ, that is, PPARγ1 and PPARγ2. PPARγ1 is present ubiquitously, whereas PPARγ2 expression is largely limited in adipocytes [7]. Based on the insulin sensitizing effects of PPARγ activation, various synthetic PPARγ agonists, which include rosiglitazone, were developed as anti-diabetic agents. These agents are classified as thiazolidinediones because of their common structural characteristics. However, the long-term administration of rosiglitazone was later found in an ADOPT study to increase susceptibility of bone fracture, especially in postmenopausal women [8–11]. Several mechanisms have been reported to explain this side effect, such as, that involving the pro-adipogenic and anti-osteoblastic effects of rosiglitazone [12, 13]. Nevertheless, it appears that the detrimental effects of rosiglitazone on bone metabolism are a consequence of its multiple effects, which include osteoblast apoptosis, the inhibition of osteoblast differentiation, or the stimulation of osteoblast differentiation and subsequent enhanced osteoblast apoptosis [14]. In particular, PPARγ2 activation by rosiglitazone suppresses the expression of Runx2, a transcription factor essential for osteoblast differentiation [15], whereas on the other hand, rosiglitazone stimulates osteoclast activities and differentiation via PPARγ-mediated c-fos activation [16].

Prolyl hydroxylase domain proteins (PHDs) play key roles in the regulation of hypoxia-inducible factor-1α (HIF-1α) under normoxia by hydroxylating two proline residues (pro-402, pro-564) in its α subunit [17, 18]. Subsequently, prolyl hydroxylated HIF-1α is recognized by von Hippel-Lindau protein (VHL), subjected to ubiquitination followed by proteosomal degradation [19–21]. So far, three PHD isoforms (PHD1, 2, and 3 also called EGLN 2, 1, and 3, respectively) have been identified in mammalian cells, and shown to have different mRNA abundances [22], substrate specificities, and inducibilities [17]. Furthermore, PHDs were recently reported to participate in myotube and adipocyte differentiation [23, 24], and dimethyloxalyl glycine (DMOG), a PHD inhibitor, was found to cause osteoblasts to adopt adipocytic phenotypes under normoxic conditions [25].

Previously, we reported that rosiglitazone induces adipocyte differentiation via PHD induction, which is followed by the ubiquitination and degradation of anti-adipogenic proteins [26]. Since there exits an inverse relationship between adipocyte and osteoblast differentiation, we sought to determine whether PHD isoforms are also involved in the suppression of osteoblast differentiation by rosiglitazone.

## Materials and Methods

### Materials

Minimum Essential Medium alpha (MEMα), fetal bovine serum (FBS), penicillin and streptomycin were obtained from GIBCO (Grand Island, NY). Rosiglitazone, Alizarin red, MG-132, protein A/G agarose, DMOG, ethyl-3,4-dihydroxybenzoate (EDHB), BADGE, GW9662 and all other chemicals were from Sigma (St Louis, MO). Antibodies against PHD1, PHD2, and PHD3 were from Novus Biologicals (Littleton, CO), and anti-PHD3 antibody for immunohistochemistry was from Abcam (Cambridge, MA). Antibodies against Runx2, goat anti-rabbit IgG, anti-mouse IgG, and mouse anti-β-actin were from Santa Cruz Biotechnology (Santa Cruz, CA). Ubiquitin, polyubiquitinated antibody, and actin were from Cell Signaling Technology (Beverly, MA).

### Animals

Female ICR mice (6 weeks of age) were obtained from Orient Bio Inc. (Seongnam, Korea). Animals were housed under specific pathogen-free conditions in an air conditioned room at 23 ± 2°C. Food and water were supplied *ad libitum*. All animal procedures were performed in accordance with the Guide for the Care and Use of Laboratory Animals published by the US National Institute of Health (National Academy, 1996), and were approved by the Animal Care and Use Committee at Gachon University.

### Cell culture and osteoblastic differentiation

Mouse primary bone marrow cells were obtained from femurs and tibias of 6-week-old ICR female mice. Mice were sacrificed by cervical dislocation, and the ends of the femurs and tibiae were cut. The bone marrow cavities of tibiae and femora were extracted with phosphate buffered saline (PBS, 5 mL) containing 1% penicillin/streptomycin and 2% FBS. The cells were cultured in MEMα complete media containing 10% heat-inactivated FBS and 100 U/mL penicillin in a 100 mm culture dish in a humidified 5% CO_2_ atmosphere at 37°C, and were allowed to adhere to the plastic support for 48 h before the first medium change. After 2 days, nonadherent cells were removed by flushing with DPBS and the medium was replaced every 2 days. Cells in passage 2 were used for the experiments. For osteogenic differentiation, primary bone marrow cells were plated at density of 7 × 10^5^ cells/well on 60 mm culture plates. After 2 days of incubation at which 90% confluency was achieved, the cells were cultured for an additional 12 days in osteogenic differentiation medium (DAG) consisting of MEMα supplemented with 10% FBS, 100 nM dexamethasone, 10 mM β-glycerophosphate, 50 μM ascorbic acid, 100 units/mL penicillin and 100 μg/mL streptomycin in the presence or absence of the indicated concentrations of rosiglitazone.

### Alizarin red staining

The mineralization of osteoblast cells was evaluated by staining with Alizarin red. Briefly, cells were washed twice with PBS, fixed with 10% formalin in PBS for 30 min, washed twice again with PBS, and then stained with Alizarin red solution (0.14 g of Alizarin red dissolved in 10 ml of distilled water and pH adjusted to ~4.2 with 30 μl ammonium hydroxide) for 20 min. Osteoblast mineralization was observed by light phase contrast microscopy.

### RT-PCR

Total RNA was isolated from cells using the easy-BLUE Total RNA extraction kit (iNtRON Inc. Korea). Reverse transcription of total RNA (1 μg) was performed using AccuPower RT PreMix (Bioneer Inc. Korea). PCR primers for PHD1, 2, and 3, Runx2, and GAPDH (internal standard) were as follows: sense, PHD1 (R)—CCG TCG GTC AGA CCA GAA AAT, PHD1 (F)—TGC CTT GCA TGC GGT ACT ATG; PHD2 (R)—CCT TGT TTC GTG TCC AGA TGG, PHD2 (F)—TGT CCG TCA CGT TGA TAA CCC; PHD3 (R)—CAC TTC CAA CCC CAT CTC CC, PHD3 (F)—CGA AGG GGT ACC CTC CAA AC; Runx2 (R)—TTC ATA ACA GCG GAG GCA TT, Runx2 (F)—CCG TGG CCT TCA AGG TTG T; GAPDH (R)—GGC ATG GAC TGT GGT CAT GA, GAPDH (F)—TTC ACC ACC ATG GAG AAG GC. Reverse transcription-PCR conditions were 35 cycles of denaturation at 94°C for 1 min, annealing at 60°C for 1 min, and extension at 72°C for 1 min, followed by a 10 min extension at 72°C. After amplification, PCR reaction mixtures were electrophoresed in 1.5% agarose gel and visualized using Gel red (Elpis biotech, Seoul) and UV irradiation. The relative abundances of mRNA were normalized versus GAPDH.

### Western blot analysis

Cells were harvested in lysis buffer containing 50 mM HEPES (pH 7.0), 250 mM NaCl, 5 mM EDTA, 0.1% Nonidet P-40, 1 mM phenylmethylsulfonyl fluoride, 0.5 mM dithiothreitol, 5 mM Na fluoride, 0.5 mM Na orthovanadate, 5 μg/ml leupeptin, and 5 μg/ml aprotinin, and incubated for 10 min at 4°C. Whole cell lysates (30 μg of protein) were loaded into an 8 or 10% sodium dodecyl sulfate-polyacrylamide gel, and then transferred to nitrocellulose membranes (Amersham Biosciences, Piscataway, NJ). Blocked membranes were then incubated with anti-PHD1, PHD2, or PHD3, and blotted with secondary antibodies conjugated with horseradish peroxidase (HRP). For immunoprecipitation, precleared lysates (100 μg of extracts) were incubated with Runx2 antibody or negative control normal mouse or rabbit IgG for 2 h at 4°C, and then Protein A/G plus agarose beads (50 μl) were added (Santa Cruz Biotechnology, CA). After overnight incubation at 4°C with constant agitation, immunoprecipitated materials were eluted with SDS-PAGE loading buffer, heated for 5 min, fractionated by SDS-PAGE, and subjected to western blot analysis. The blot was incubated with anti-ubiqutin antibody for overnight at 4°C. After washing with TBS-T, blots was then incubated with secondary antibodies conjugated with horseradish peroxidase (HRP). Immunoreactive bands were visualized by enhanced chemiluminescence (Amersham Life Science, Buckinghamshire, UK), and band densities were quantified using UN-SCAN-IT gel ver. 5.1 (Silk Scientific, Inc., Orem, UT) and normalized versus β-actin. Protein concentrations were determined using Bio-Rad protein assay reagent according to the manufacturer’s instructions.

### PHD knockdown and treatment with PHD inhibitors

Transfection of short hairpin RNA (shRNA, 1 μg each/well) against PHD1 (sc-45617-SH) and PHD2 (sc-45538-SH), or siRNA (20 μM each/well) against PHD3 (sc-45800), or Control (Santa Cruz, CA) into primary bone marrow cells (2 x 10^5^ cells/well at 80–90% confluence) was accomplished using Lipofectamine 2000 reagent in OPTI-MEM (Invitrogen) medium. Six hours after transfection, the medium was replaced with MEMα medium containing 10% FBS, and the cells were treated with or without rosiglitazone. Knockdowns were carried out twice on day 0 and day 4 of a 12-day differentiation period.

### PHD inhibitors and PPARγ antagonist treatment

PHD inhibitors (1 mM DMOG or 20 μM EDHB) or PPARγ antagonists (20 μM BADGE or 1 μM GW9662) were added to primary bone marrow cells at DAG induction and maintained in medium by changing the medium every 3 days in the presence of rosiglitazone. Inhibitors and antagonists were pretreated for 1 h and then 10 μM rosiglitazone was added. DMSO was used as a solvent for each reagent.

### *In vivo* study

ICR female mice (6 weeks of age) were orally administered either vehicle (saline, n = 10) or rosiglitazone (10 mg/kg/day, n = 10) for 8 weeks. After 8 weeks of treatment, tibiae and femora were isolated and bone marrow was extracted. Immediately after extraction, bone marrow cells were seeded on 12-well plates (9 x 10^6^ cells/well) and cultured until 90% confluent. Separately, whole right femora of mice were harvested, and bone mineral densities (BMD), bone mineral contents, and femoral lengths were measured by peripheral dual-energy X-ray absorptiometry (pDEXA) using a pDEXA scanner (SabreTM; Norland Medical Systems Corp., Fort Atkinson, WI, USA) and pDEXA Sabre software (version 3.9.4; Norland Medical Systems Corp.). Scans (4 × 4 cm) of entire femora were performed at 20 mm/s and a resolution of 0.2 × 0.2 mm. BMD values were computed by the software as a function of dual beam attenuation (28 and 48 KeV) of X-rays generated by the sources and traveled along a defined area above the specimen. BMD histogram averaging width was set to 0.01 g/cm^2^ for all bone scans. *In vitro* precision was expressed as the coefficient of variation (CV = 100 × standard deviation/mean) and was calculated by measuring the BMD of a phantom of density 0.929 g/cm^2^.

For immunohistochemistry, femora of mice were dissected and specimens were fixed in 10% buffered formalin for 48 h, decalcified with 10% EDTA (pH 7.0) for 3–4 weeks at 4°C, and embedded in paraffin. For immunostaining, antigen retrieval of tissue sections (5 μm for bone) was performed using a domestic microwave in Tris/EDTA buffer (pH 9.0) for 2–3 min. Sections were incubated with antibodies against PHD1, 2, 3 and Runx2 at 25°C for 2 h, and then incubated for 30 min with horseradish peroxidase–conjugated secondary antibodies attached to anti-rat or anti-rabbit immunoglobulin G. Antibody complexes were detected using 3, 3′-diaminobenzidine tetrahydrochloride (Dako, Glostrup, Denmark). Sections were subsequently counterstained with hematoxylin (Dako), and photographed using a Zeiss Axio Imager Z1 microscope (Carl Zeiss) to allow histomorphometric measurements of central areas of metaphyseal bone to be made on digitalized images, which were captured using a Mirax Desk scanner (Carl Zeiss). Staining intensity of PHD1, PHD2 and PHD3 was analyzed using color deconvolution feature of the ImageJ software.

### Statistical analysis

Results are expressed as means ± SEMs. Statistical significances were determined by one-way analysis of variance (ANOVA) followed by Tukey’s multiple-comparison test. *P* values of < 0.05 were considered statistically significant.

## Results

### Effects of rosiglitazone on osteoblast differentiation

**Fig 1:**
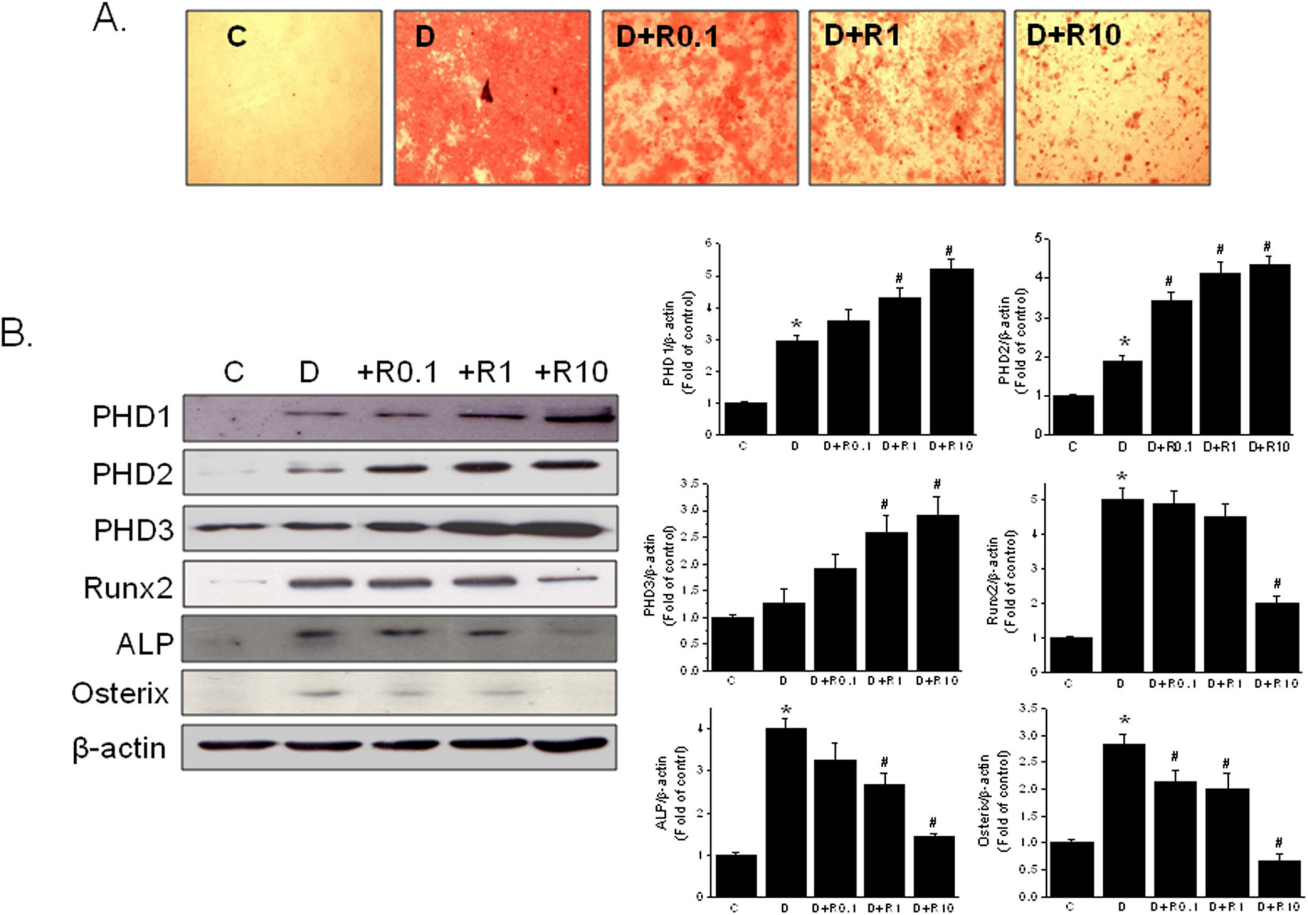
Concentration dependent effects of rosiglitazone on osteoblast differentiation. Primary bone marrow cells were treated with the indicated concentrations of rosiglitazone for 12 days in the presence of DAG and extents of osteoblast differentiation were determined by Alizarin red staining (A) and western blotting (B). Experiments were conducted three times and representative results are shown. Densitometric analysis was conducted using UN-SCAN-IT gel ver. 5.1 software (Silk Scientific), and is expressed as means ± SEMs of three experiments. *P<0.05 vs control (C), #*P*<0.05 vs differentiated (D). C, control (no differentiation); D, differentiated in DAG medium; R, rosiglitazone.

The experiment was conducted using primary bone marrow cells isolated from female ICR mice. Rosiglitazone concentration dependently (0.1–10 μM) suppressed osteoblast differentiation induced by DAG (Fig 1A), as assessed by Alizarin red staining. As was shown previously [26], rosiglitazone induced the expression of PHD1, 2, and 3 by 3- to 5-fold; PHD3 induction was lowest although the basal level of PHD3 was high (Fig 1B). Consistent with the suppressive effects of rosiglitazone on osteoblast mineral deposition (Fig 1A), the level of the osteoblast marker Runx2, ALP and osterix was concentration-dependently reduced in response to rosiglitazone; at 10 μM, rosiglitazone markedly reduced all osteogenic markers such as Runx2, ALP and osterix levels (Fig 1B). Cell viabilities were similar in the rosiglitazone- and vehicle-treated groups.

**Fig 2:**
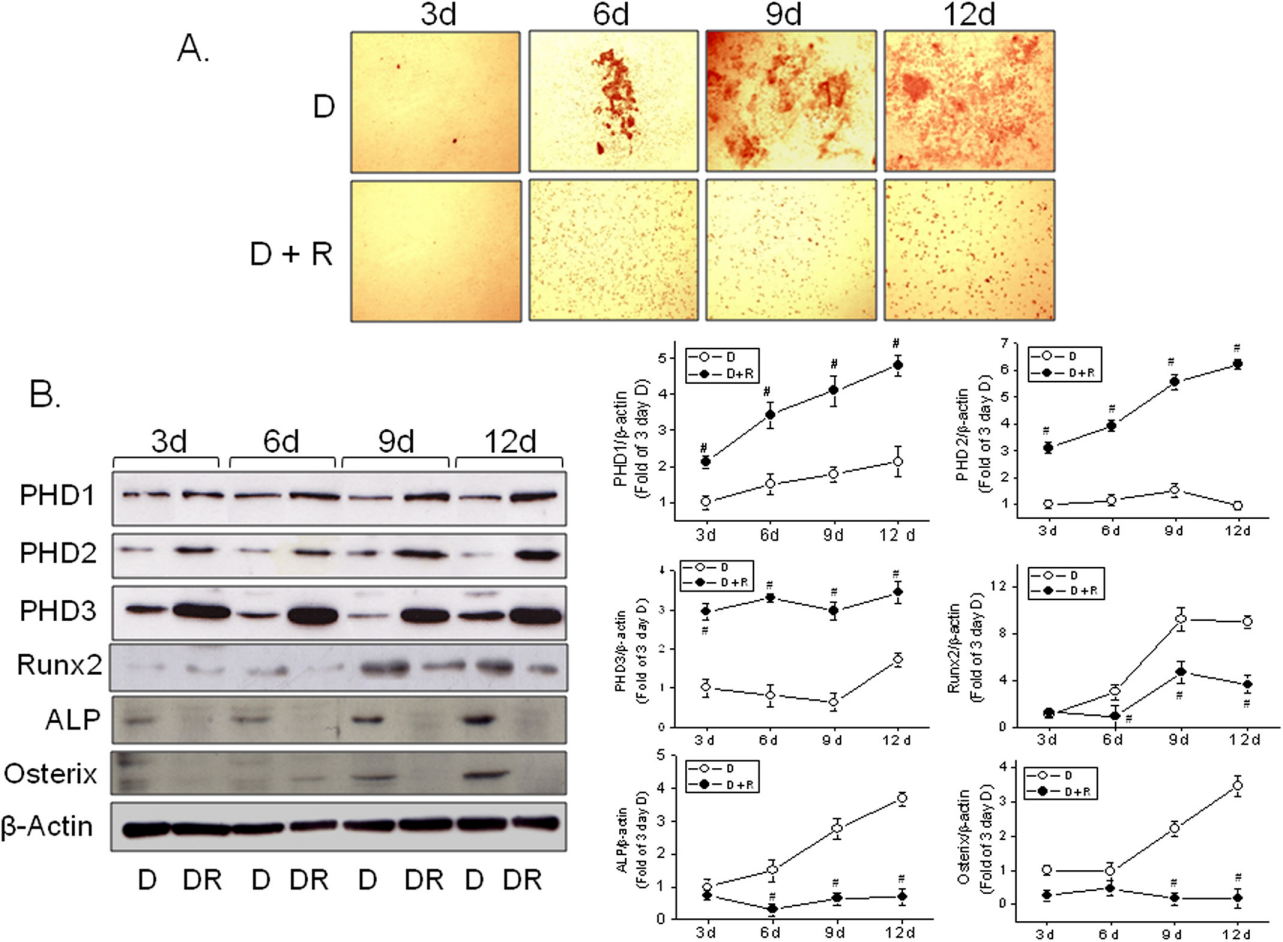
Time dependent effects of rosiglitazone on osteoblast differentiation. Primary bone marrow cells were treated with rosiglitazone (10 μM) at the indicated times in the presence of DAG. Extents of osteoblast differentiation were determined by Alizarin red staining (A) and western blotting (B). Experiments were carried out three times and representative results are shown. Densitometric analysis was conducted using UN-SCAN-IT gel ver. 5.1 software (Silk Scientific) and is expressed as means ± SEMs of three experiments. #*P*<0.05 vs differentiated (D). D, differentiated in DAG medium; DR, DAG in the presence of rosiglitazone.

Next, we examined the temporal effects of rosiglitazone on osteoblast differentiation. As shown in Fig 2A, osteoblast differentiation was evident at 6 days after DAG induction, and rosiglitazone (10 μM) suppressed osteoblast differentiation. Similarly, the induction of PHD isotypes by rosiglitazone was detected even at 3 days after induction, and isotype levels remained at 12 days after induction (Fig 2B). Consistent with previous findings [14, 15], Runx2, ALP and osterix expression was enhanced along with osteoblast differentiation, and this was markedly diminished in the presence of rosiglitazone (10 μM) on day 12 (Fig 2B).

### Effects of PHD inhibitors on rosiglitazone action

**Fig 3:**
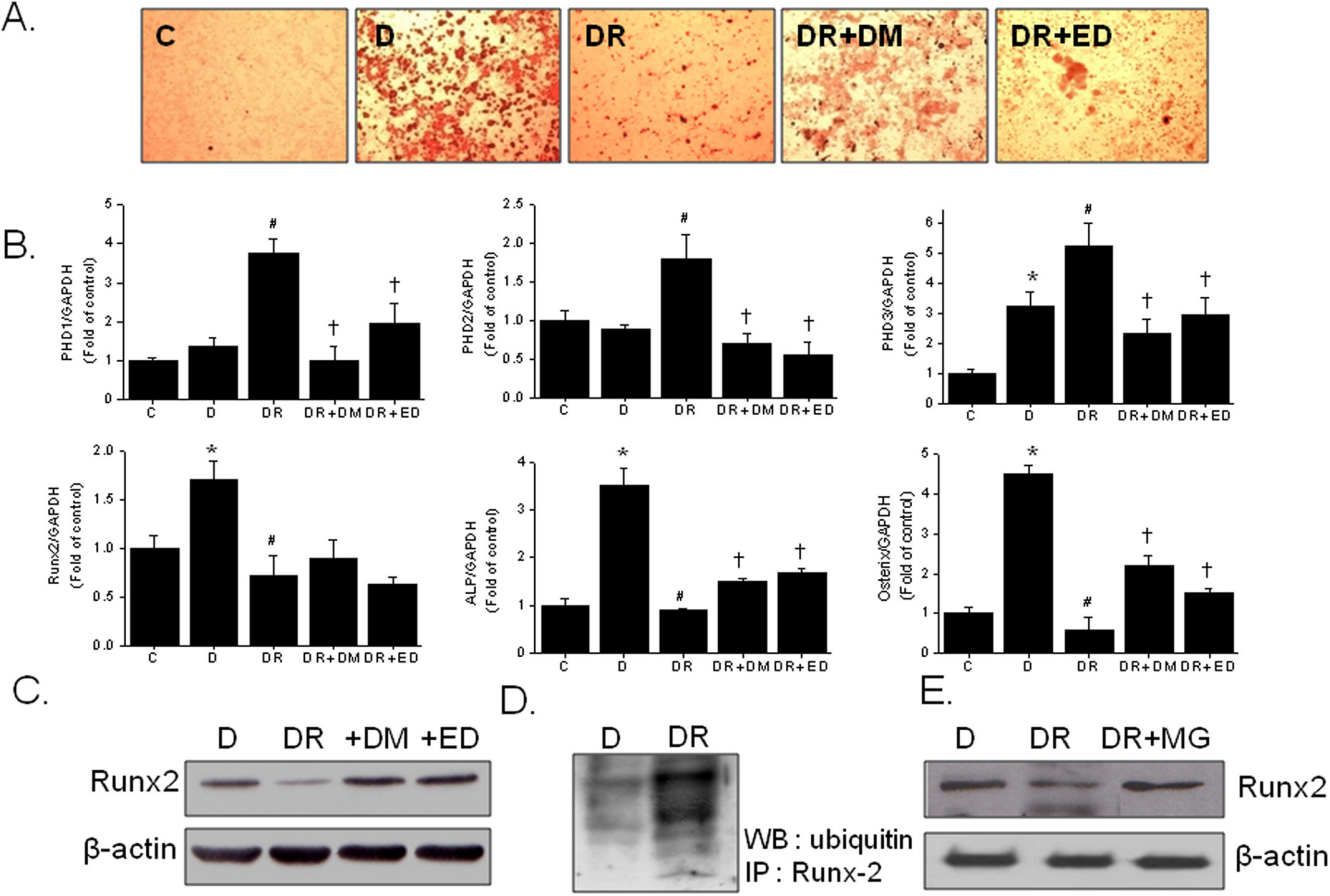
Effects of PHD inhibitors on rosiglitazone-induced anti-osteoblast differentiation. Primary bone marrow cells were treated with either DMOG (1 mM) or EDHB (20 μM) in the presence or absence of rosiglitazone (10 μM) for 12 days (media were changed every 48 h). Extents of osteoblast differentiation were monitored by Alizarin red staining (A), and the mRNA levels of the PHD 1–3 and osteomarker marker genes were determined by RT-PCR (B). The protein levels of Runx2 and levels of Runx-2 ubiquitination were determined by western blotting (C) and by immunoprecipitation using polyubiquitination antibody (D). The effect of MG-132 (5 μM; an inhibitor of proteasomal degradation) on Runx-2 expression was examined by western blotting (E). Experiments were conducted three times and representative results are shown. Densitometric analysis was conducted using UN-SCAN-IT gel ver. 5.1 software (Silk Scientific) and is expressed as means ± SEMs of three experiments. *P<0.05 vs control (C), #*P*<0.05 vs differentiated (D), ✝*P*<0.05 vs DR. C, control (no differentiation); D, differentiated in DAG medium; DR, DAG in the presence of rosiglitazone; DM, DMOG; ED, EDHB; MG, MG-132.

To elucidate the roles of PHD isotypes during the suppression of osteoblast differentiation by rosiglitazone, we examined the effects of two well-known PHD inhibitors (DMOG and EDHB) on the effects of rosiglitazone. Both inhibitors prevented the suppression of osteoblast differentiation by rosiglitazone (Fig 3A), and reduced the mRNA expressions of PHD isotypes, which suggests that the regulation of PHD expression by rosiglitazone occurs at the transcriptional level, and that PHD induction is probably related to the inhibition of osteoblast differentiation by rosiglitazone (Fig 3B). Furthermore, the mRNA expression of Runx2, ALP and osterix was increased during osteoblast differentiation, and rosiglitazone had an inhibitory effect on the mRNA expression of osteogenic markers. The PHD inhibitors had little or partial effect on the rosigltiazone-reduced mRNA expression of osteogenic markers.

Since PHDs are known to play roles in protein degradation via ubiquitination, we next examined if PHDs are involved in the degradation of Runx2, a well-known transcription factor involved in osteoblast differentiation. As shown in Fig 3C, reduced levels of Runx2 protein by rosiglitazone were prevented in the presence of PHD inhibitors. Noticeably, the extent of ubiquitination of Runx2 was dramatically enhanced after rosiglitazone treatment, while MG-132, a proteasomal degradation inhibitor restored Runx2 protein level to differentiated control group (Fig 3D and 3E). Although the transcriptional suppression of Runx2 by PPARγ activation might also contribute to the inhibitory effect of rosiglitazone on osteoblastogenesis, these results suggest that posttranslational modification of Runx2 by PHDs and the subsequent stimulated ubiquitination of Runx2 could play a significant role in the suppression of osteoblast differentiation by rosiglitazone.

### Effects of PHD isotype knockdown on rosiglitazone action

**Fig 4:**
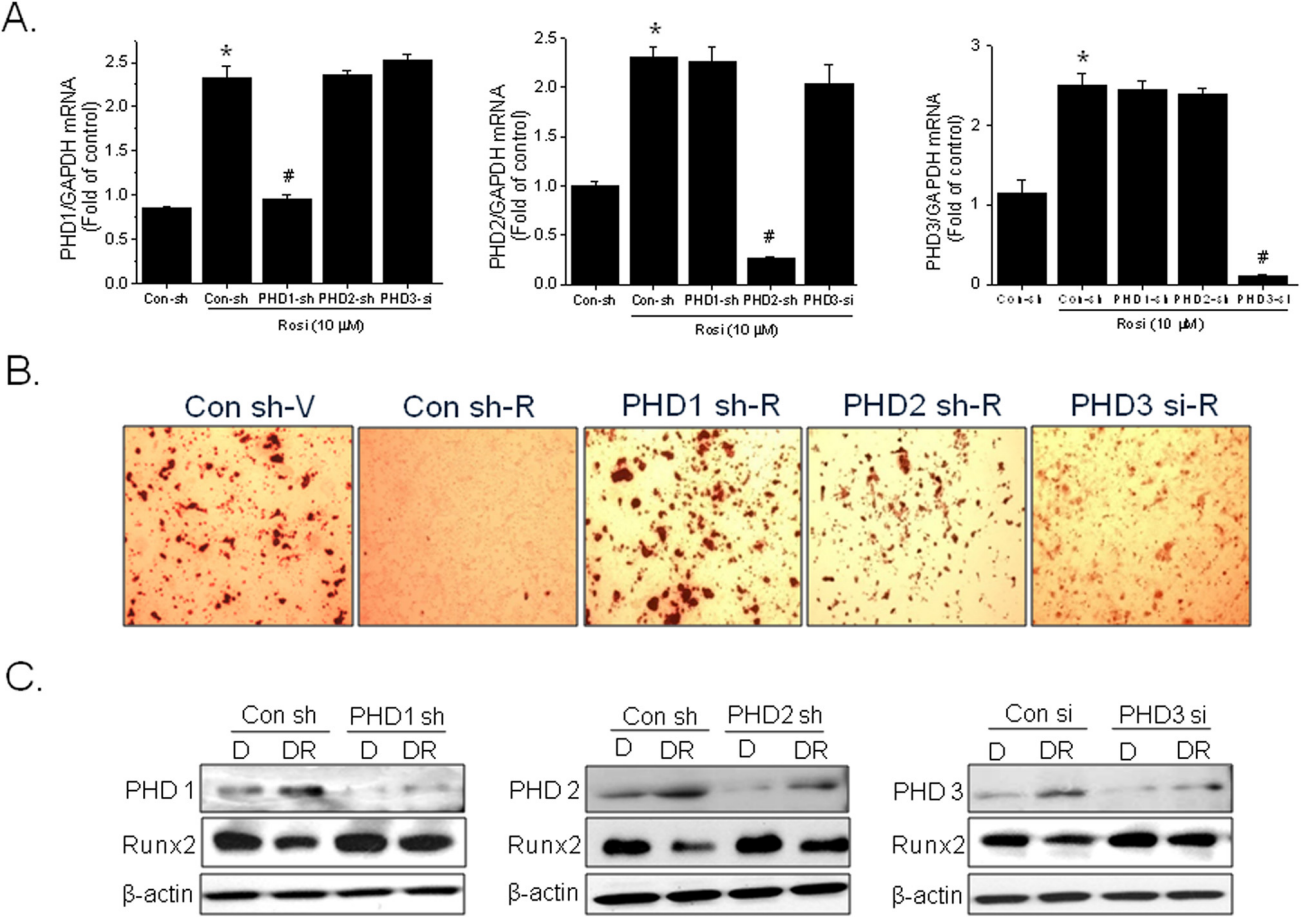
Effects of PHD knockdown on the rosiglitazone-induced inhibition of osteoblast differentiation. The PHD1, 2, and 3 isoforms were knocked down by transfecting in primary bone marrow cells with isoform-specific shRNAs or siRNA on day 0 and day 4 of a 12-day differentiation period. Extents of knockdown were determined by RT-PCR (A), and osteoblast differentiation was monitored by Alizarin red staining (B). The protein levels of Runx2 were determined by western blotting (C). Experiments were conducted twice and representative results are shown. Densitometric analysis was conducted using UN-SCAN-IT gel ver. 5.1 software (Silk Scientific) and is expressed as means ± SEMs of two experiments. *P<0.05 vs control shRNA in the absence of rosiglitazone, #*P*<0.05 vs control shRNA in the presence of rosiglitazone.

To determine whether certain PHD isoform(s) is responsible for the suppression of osteoblast differentiation by rosiglitazone, each PHD1, 2, and 3 isoform was knocked down by shRNA transfection in primay bone marrow cells. PHD1 and PHD2 shRNA specifically knocked down the expression of the respective PHD isotypes in a specific manner whereas PHD3 shRNA also knocked down PHD2 mRNA (25% inhibition). PHD3 siRNA was alternatively used to specifically knockdown PHD3 mRNA expression without interfering PHD2 expression (Fig 4A). Knockdown of each PHD isoform blocked the rosiglitazone-induced suppression of osteoblast differentiation (Fig 4B), along with increased Runx2 protein levels (Fig 4C). These results suggest that PHD induction is responsible for the inhibitory effect of rosiglitazone on osteoblast differentiation, and that each PHD isoform may be involved in rosiglitazone action.

### Effects of PPARγ antagonists on rosiglitazone action

**Fig 5:**
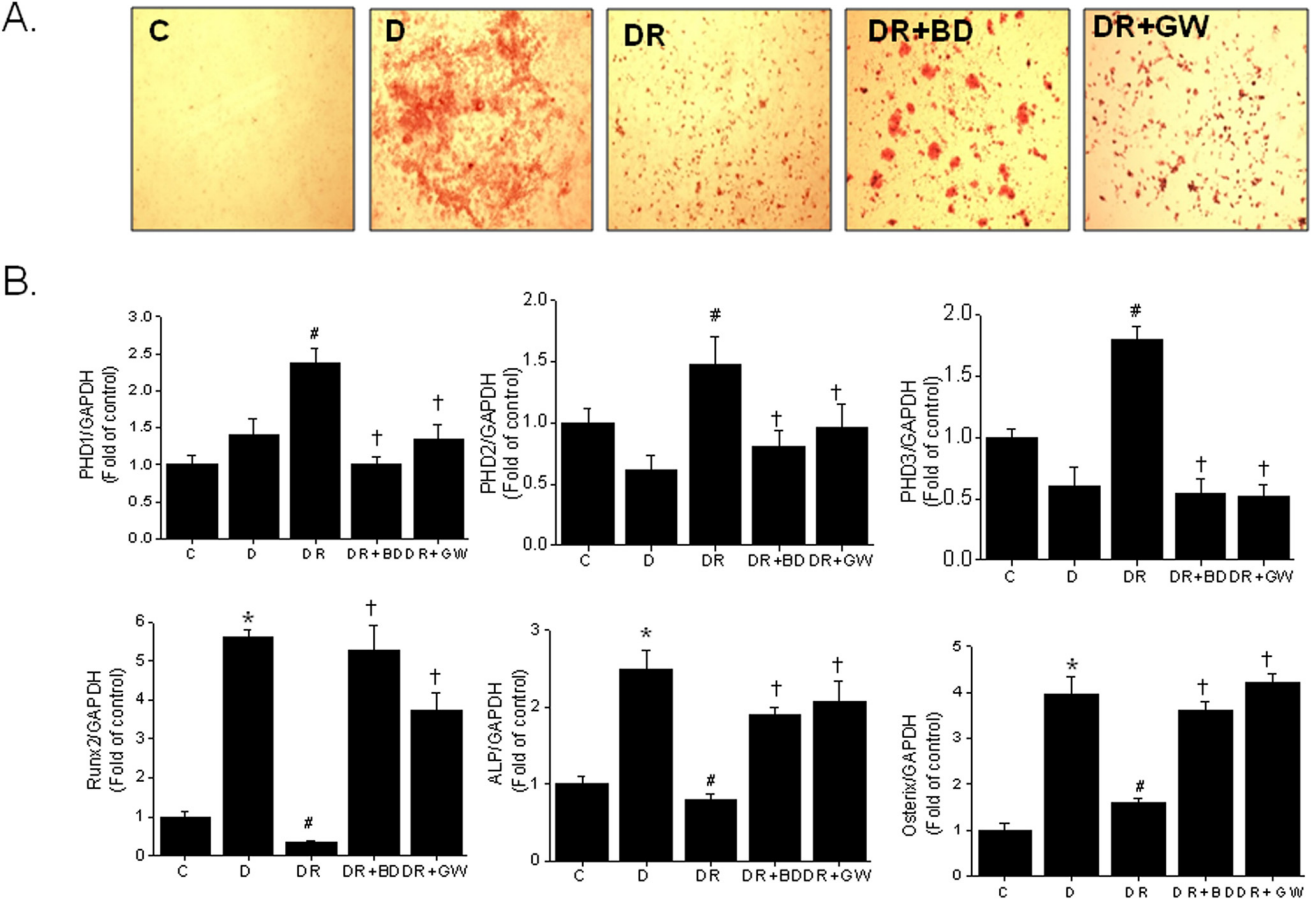
Effects of PPARγ antagonists on rosiglitazone-induced adipocyte differentiation. Primary bone marrow cells were treated with BADGE (20 μM) or GW9662 (1 μM) in the presence of rosiglitazone (10 μM) for 12 days. Osteoblast differentiation was determined by Alizarin red staining (A), and the mRNA levels of PHD1, 2, and 3, and osteogenic marker genes were analyzed by RT-PCR (B). Densitometric analysis was conducted using UN-SCAN-IT gel ver. 5.1 software (Silk Scientific) and is expressed as means ± SEMs. *P<0.05 vs control (C), #*P*<0.05 vs differentiated (D), ✝*P*<0.05 vs DR. Experiments were conducted three times and representative results are shown. C, control (no differentiation); D, differentiated in DAG medium; DR, DAG in the presence of rosiglitazone; BD, BADGE; GW, GW9662.

To determine whether the effect of rosiglitazone on osteoblast differentiation is mediated by PPARγ, we examined the effects of two well known PPARγ antagonists, that is, BADGE and GW9662. Both antagonists partially inhibited the rosiglitazone-induced suppression of osteoblast differentiation (Fig 5A), and inhibited rosiglitazone-induced PHD isotype expressions (Fig 5B). As shown in Fig 5B, the mRNA expression of Runx2, ALP and osterix decreased when primary bone marrow cells were treated with rosiglitazone, and this decrease prevented by the presence of BADGE or GW9662. Together, these results suggest that rosiglitazone-induced PHD upregulation results from the activation of PPARγ by rosiglitazone.

### *In vivo* effects of rosiglitazone

To examine whether the effects of rosiglitazone observed at the cellular level occur in *in vivo*, rosiglitazone (10 mg/kg) was administered orally to ICR female mice once daily for 8 weeks. A slight increase in body weight and an increase in fat mass were observed in rosiglitazone-treated mice as compared with vehicle-treated mice (Table 1). DEXA analysis of excised femora revealed that bone mineral density and bone mineral content were decreased by rosiglitazone as compared with vehicle controls although statistically insignificant (0.0580 ± 0.0027 vs 0.0643 ± 0.0019 g/cm^2^, respectively, for BMD; 0.0212 ± 0.0012 vs 0.0233 ± 0.0008 g, respectively, for BMC). Furthermore, rosiglitazone had decreased tendency of widths and lengths of femora. In addition, plasma levels of ALP were significantly decreased by rosiglitazone (Table 2), supporting the suppressive action of rosiglitazone on bone metabolism. Consistent with previous results, plasma triglyceride was decreased by rosiglitazone, but fasting plasma glucose levels were not affected after treatment (results not shown).

**Table 1:** DEXA analysis of rosiglitazone-treated mice.

Factors	Vehicle group (n = 10)	Rosiglitazone group (n = 10)
**BMD (g/㎠)**	0.0643 ± 0.0019	0.0580 ± 0.0027
**BMC (g)**	0.0233 ± 0.0008	0.0212 ± 0.0012
**Area (㎠)**	0.3617 ± 0.0048	0.3635 ± 0.0053
**Length (㎝)**	1.6500 ± 0.0153	1.5500 ± 0.1029
**Width (㎝)**	0.4150 ± 0.0065	0.3950 ± 0.0085
**Lean mass (g)**	0.0960 ± 0.0003	0.0106 ± 0.0003
**FAT mass (g)**	0.0210 ± 0.0006	0.0231 ± 0.0006
**Body weight (g)**	24.75 ± 0.35	25.87 ± 0.33

**Table 2:** Blood analysis of rosiglitazone-treated mice.

Factors	Vehicle group (n = 10)	Rosiglitazone group (n = 10)
**ALP (U/L)**	69.4 ± 3.89	42.6 ± 7.81*
**T-CHO (mg/dl)**	102 ± 9.83	82.8 ± 3.21
**TG (mg/dl)**	175 ± 12.2	105 ± 9.09*
**HDL (mg/dl)**	59.2 ± 5.98	47.6 ± 2.41
**LDL (mg/dl)**	16.4 ± 1.14	14.5 ± 0.85

*P<0.05 vs vehicle group

**Fig 6:**
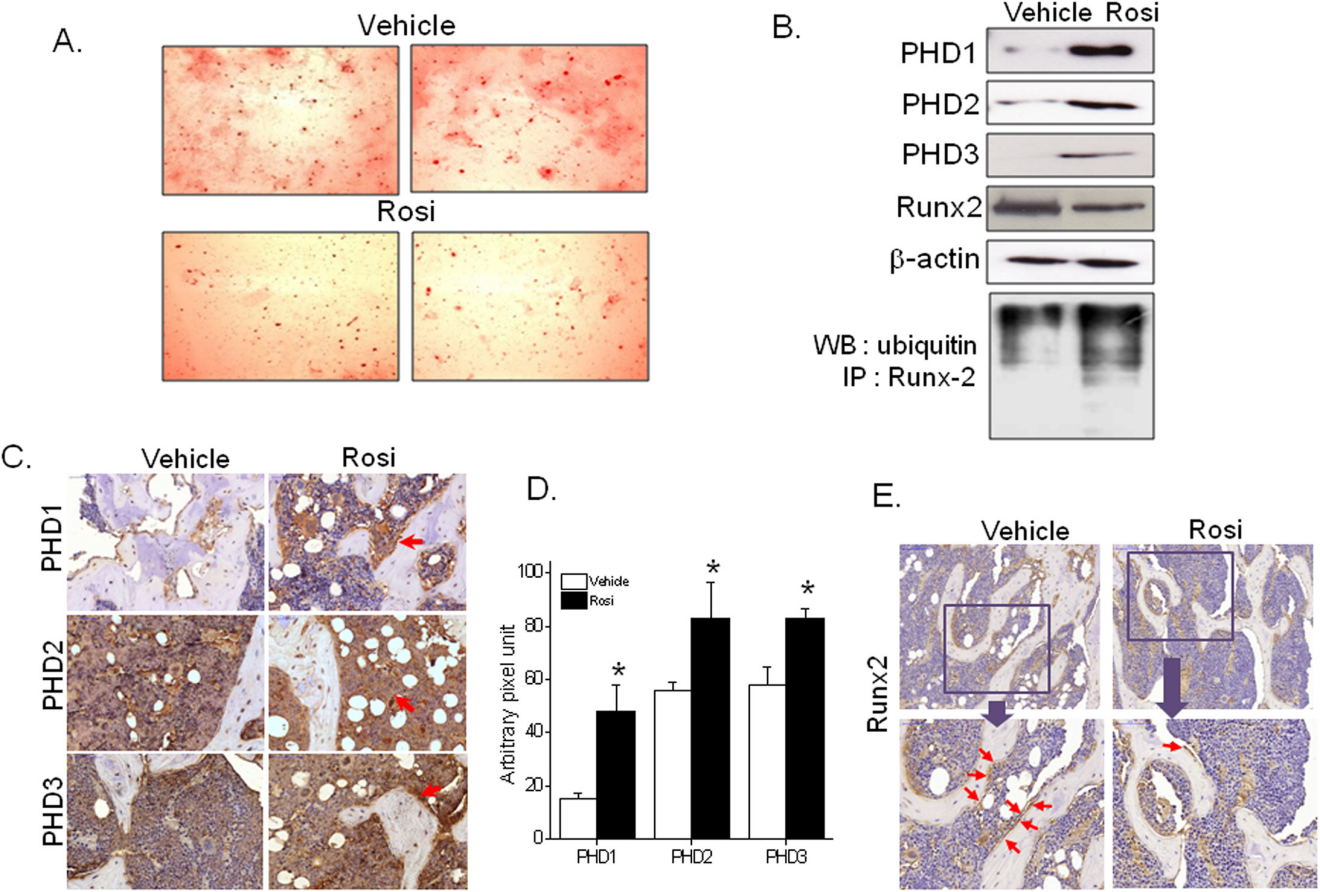
*In vivo* effects of rosiglitazone in female ICR mice. Rosiglitazone (10 mg/kg once daily) was administered orally to ICR mice (6 weeks old) for eight weeks. Primary bone marrow cells were isolated from the femora of both vehicle and rosiglitazone-treated animals, and extents of osteoblast differentiation were determined by Alizarin red staining (A). PHD expression and ubiquitinated Runx2 levels were determined by western blotting and immunoprecipitation, respectively (B). Immunohistochemistry of the PHD isoforms (C), and quantification of PHD1, PHD2, and PHD3 signal intensity in bone marrow section (D). Runx2 expression was determined by immunohistochemistry (E). Red arrow indicates each PHD and Runx2 expression. Experiments were conducted three times and representative results are shown. *P<0.05 vs vehicle.

The extent of osteoblast differentiation was determined *ex vivo* using bone marrow cells isolated from the femora of rosiglitazone-treated ICR mice, and it was found rosiglitazone decreased osteoblast differentiation (Fig 6A). The stimulatory effects of rosiglitazone on PHD expression were confirmed by western blot (Fig 6B) and immunohistochemistry (Fig 6C and 6D). As was expected from the results of the *in vitro* study, the protein level of Runx2 was reduced by rosiglitazone (Fig 6B and 6E), and its ubiquitination was enhanced (Fig 6B). These results support our *in vitro* findings that rosiglitazone suppresses osteoblast differentiation via PPARγ-mediated PHD induction and the subsequent ubiquitination and degradation of Runx2.

## Discussion

The present study shows that rosiglitazone suppresses osteoblast differentiation via PPARγ-mediated PHD upregulation, which in turn leads to increased ubiquitination and degradation of Runx2, an essential transcription factor for osteoblast differentiation. Furthermore, the *in vivo* oral administration of rosiglitazone to mice decreased osteoblast differentiation and increased the expression of PHD in primary bone marrow cells isolated from femurs and this was associated with reduced bone mineral density. Collectively, our results show that PHD induction by rosiglitazone accounts, at least in part, to the suppression of osteoblast differentiation by rosiglitazone.

Rosiglitazone is a well-known insulin sensitizer, and as such has been used for the treatment of type 2 diabetes mellitus. However, despite its efficacious effects, the long term use of rosiglitazone is limited by its negative effect on bone quality [11, 27, 28]. Although the detrimental effects of rosiglitazone on bone quality are well recognized, the mechanism responsible has yet to be elucidated. In humans, studies show that thiazolidinedione (including rosiglitazone)-induced bone loss is attributable to reduced bone formation [9] and increased bone resorption [12], both of which are required for the uncoupling of bone remodeling. In terms of the action of rosiglitazone on osteoblast differentiation, previous results are discrepant, for example, some have shown rosiglitazone has no detectable effect on osteoblast differentiation or osteoblast number [29, 30], and other that rosiglitazone stimulates osteoblast differentiation and subsequent apoptosis [15, 31]. On the other hand, rosiglitazone has been reported to have a direct negative effect on osteoblast numbers and functions [32, 33], which concur with our results regarding the inhibitory effect of rosiglitazone on osteoblast differentiation, and to stimulate osteoclast differentiation [34]. However, the relative magnitudes of these effects appear to vary between studies, which suggest that the regulation of bone homeostasis by thiazolidinediones is context-dependent, that is it depends on age, sex, metabolic state, duration and dosage of thiazolidinediones, and other factors. Moreover, the effects of various PPARγ2 ligands on adipocyte and osteoblast differentiation are diverse [35], and suggest the influences of distinct regulatory pathways that depend on the nature of ligand/receptor interactions.

The roles played by PHD isoforms in cellular differentiation have been recently revealed. For example, PHD3 (EGLN3) appears to play a role in the regulation of myogenin stability [23]. In our previous study on rosiglitazone-induced adipocyte differentiation, the expressions of PHD isoforms were found to be induced by rosiglitazone in a PPARγ-dependent manner and to lead to the ubiquitination of anti-adipogenic proteins and their degradation, and these pathways appear to be independent of HIF-1α regulation [26]. Recent report on the reciprocal effects of adipocyte and osteoblast lineage commitment after thiazolidinedione treatment [36] encouraged us to further investigate the role of PHD isoforms in rosiglitazone-suppressed osteoblast differentiation. As was expected, rosiglitazone suppressed osteoblast differentiation in primary bone marrow cells and increased the expressions of PHD isoforms at the mRNA and protein levels, implying that the transcriptional regulation of PHDs by rosiglitazone might underlie the effects of rosiglitazone. Our previous ChIP-PCR analysis using C3H10T1/2 cells, suggested PPARγ can bind directly to the promoter region of each PHD [26], which further supports the notion that the regulation of PHD expression by rosiglitazone occurs at the transcriptional level. As was observed for adipocyte differentiation, PHD inhibitors and PHD shRNAs/siRNA prevented rosiglitazone inhibiting osteoblast differentiation, demonstrating the critical role played by PHD isoforms in the suppression of osteoblast differentiation by rosiglitazone. In the present study, all three isoforms behaved in a similar manner, but the relative contributions of the three isoforms remain to be determined under physiological conditions.

Several studies on the effects of PHD on osteoblast differentiation have produced conflicting results. PHD inhibitors under normoxic conditions inhibited osteoblast gene expression [25], suggesting that PHD is critical for regulation of the osteoblast phenotype. In contrast, DMOG treatment of human mesenchymal stem cells under normoxic conditions increased osteogenesis in a HIF1α dependent manner [37, 38]. On the other hand, PHD2 appears to be involved in ascorbic acid-induced osterix expression via the ubiquitination-mediated degradation of unidentified transcription repressors of osterix [39]. The basis of the discrepancies regarding the roles of PHD isoforms in osteoblast differentiation remains unclear, which is possibly due to the use of mesenchymal stem cells from different sources, different culture conditions, and different experimental designs.

Runx2 is a member of the Runx family and shares structural similarities with other family members [40]. Lecka-Czernik et al. [15] reported that the downregulation of Runx2 underlies the PPARγ-dependent suppression of osteoblastogenesis and the enhancement of adipogenesis by rosiglitazone. Since rosiglitazone induced PHD isoforms at the time of suppressing osteoblastogenesis under our experimental conditions, we hypothesize that Runx2 is targeted by PHD isoforms. It has been shown cellular levels of Runx are controlled by various posttranslational modifications, such as, ubiquitination [41]. Notably, Runx2 contains three activation domains (AD1-3), and the AD-3 domain contains a proline/serine/threonine (PST)-rich region near its C terminus, which is probably ubiquitinated [42]. Since in the present study, the effects of PHD inhibitors were independent of HIF-1α regulation (results not shown), the indirect effect of PHD induction, namely, HIF-1α-mediated Runx2 regulation, is unlikely to be responsible for the anti-osteoblastogenetic effect of rosiglitazone [43]. Previous reports have shown that Runx2 mRNA expression is reduced in early progenitor cells by rosiglitazone [31], and we also noted rosiglitazone had suppressive effect on Runx2 mRNA expression, which suggests an additional mechanism of Runx2 suppression by rosiglitazone. Further investigations are required to delineate the natures of interactions between Runx-2 and PHD isoforms, as they could identify new modifications of Runx2.

To achieve *in vivo* evidence of PHD involvement in rosiglitazone-suppressed bone formation, rosiglitazone was orally administered for 8 weeks to female ICR mice. Rosiglitazone was found to concomitantly reduce bone mineral density and plasma ALP activity. These findings are consistent with previous results [44], and reflect *in vivo* suppression of osteoblastogenesis by rosiglitazone. In parallel, increased PHD levels in femoral primary bone marrow cells were detected by western blot or immunohistochemistry after *in vivo* rosiglitazone administration, which supports our *in vitro* results suggesting that PHD isoforms participate in the reduced osteoblast differentiation induced by rosiglitazone. In a previous study, PHD inhibitors increased bone mineral density, bone microarchitecture, and bone mechanical strength in ovariectomized rats [45], which agrees with our observations. Of the many potential mediators of the effect of rosiglitazone on cellular differentiation, PHDs may play an indispensible role in the regulations of osteoblast and adipocyte differentiation by rosiglitazone.

Summarizing, the present study demonstrates that PHD isoforms participate in the anti-osteoblast differentiation action of rosiglitazone by regulating the stability of Runx2 in a PPARγ-dependent manner. Although effects other than the suppression of osteoblast differentiation may contribute to rosiglitazone-induced diminished bone quality, the findings of the present study have possible physiological relevance to clinically encountered rosiglitazone-induced bone loss. More importantly, the present study provides new insights regarding the developments of more effective PPARγ activators with fewer side effects.
